# Biofilm and fluoroquinolone resistance of canine *Escherichia coli* uropathogenic isolates

**DOI:** 10.1186/1756-0500-7-499

**Published:** 2014-08-07

**Authors:** Manuela Oliveira, Filipa Rocha Dias, Constança Pomba

**Affiliations:** 1 CIISA/Faculdade de Medicina Veterinária, Universidade de Lisboa, Avenida da Universidade Técnica, 1300-477 Lisbon, Portugal

**Keywords:** Biofilm, Dogs, *Escherichia coli*, Fluoroquinolone resistance, Urinary tract infection

## Abstract

**Background:**

*Escherichia coli* is the most common uropathogen involved in urinary tract infection (UTI). Virulence of strains may differ, and may be enhanced by antimicrobial resistance and biofilm formation, resulting in increased morbidity and recurrent infections. The aim of this study was to evaluate the *in vitro* biofilm forming capacity of *E. coli* isolates from dogs with UTI, by using fluorescent *in situ* hybridization, and its association with virulence genes and antimicrobial resistance.

**Findings:**

The proportion of biofilm-producing isolates significantly increased with the length of incubation time (P < 0.05). Biofilm production was significantly associated with fluoroquinolone resistance at all incubation time points and was independent of the media used (P < 0.05). Biofilm production was not associated with *cnf1*, *hly*, *pap* and *sfa* genes (P > 0.05), but was significantly associated with *afa*, *aer* and the β-lactamase genes (P < 0.05).

**Conclusions:**

To the best of our knowledge, this is the first report showing significant association between biofilm production and fluoroquinolone resistance in *E. coli* isolates from dogs with UTI. Biofilm formation may contribute to UTI treatment failure in dogs, through the development of bacterial reservoirs inside bladder cells, allowing them to overcome host immune defenses and to establish recurrent infections.

## Findings

*Escherichia coli* is the most common uropathogen in urinary tract infections (UTI) of humans and animals, being responsible for high morbidity and increased health care costs [1-3]. These infections are usually considered acute and self-limiting, but recurrent clinical signs are often observed [3]. *E. coli* UTI pathogenesis is similar in dogs and humans, and dogs may serve as reservoirs of uropathogenic *E. coli* (UPEC) strains that can be transmitted to humans and other animals [2,4]. In fact, the human highly virulent O25:ST131 uropathogenic clone was recently found in a dog with chronic cystitis [5,6]. This fact suggests a possible human-to-animal transmission.

In humans, it is well established that UPEC are able to form biofilm structures within the bladder, forming bacterial reservoirs that allow infection persistence [7-10]. These structures are highly organized multicelular complexes, characterised by adherent colonies surrounded by a large exopolysaccharide matrix. Biofilm structures protect bacteria against high antimicrobial concentrations and phagocytosis, allowing their survival in hostile environments within the host [10]. Detection of biofilm-producer strains is therefore relevant for the design of adequate control measures for UPEC infections. Fluoroquinolones are extensively used for UTI treatment, due to the high concentration levels reached in the urinary tract and good tissue concentrations [11]. The aim of this study was to evaluate the *in vitro* biofilm-forming ability of *E. coli* isolates from dog urinary tract infections, and its association with virulence and β- lactamase antimicrobial resistance genes, and with 2^nd^ generation quinolones resistance.

Sixty-six *E. coli* isolates were used, from a collection of bacterial isolates from dogs with UTI belonging to the Faculty of Veterinary Medicine, University of Lisbon. Isolates virulence factors had already been determined by multiplex PCR and described by us: 57.6% (n = 38) were positive for S fimbriae gene *sfa*; 1.5% (n = 1) for afimbrial adhesion I gene *afaI*; 42.4% (n = 28) for haemolysin gene *hly*; 40.9% (n = 27) for cytotoxic necrotizing factor I gene *cnfI*; 34.8% (n = 23) for aerobactin gene *aer*; and 42.4% (n = 28) for pyelonephritis-associated pili gene *pap*[12]. Detection of genes related with β-lactamase resistance has also been previously described by us: 19 isolates were positive for *bla*_TEM_ (28.8%), three for *bla*_SHV_ (4.5%), two for *bla*_OXA-1_ (3.0%) and six for *ampC* (9.1%) [13].

Minimum inhibitory concentrations (MIC) of ciprofloxacin (CIP, Laboratório Atral-Cipan, Portugal), enrofloxacin (ENR, Bayer, Germany), marbofloxacin (MAR, Vétoquinol, France) and orbifloxacin (OBX, Schering-Plough, USA) were determined by broth microdilution, following Clinical and Laboratory Standards Institute guidelines [14,15]. *E. coli* ATCC 25922 was used as a reference control for MIC testing. Dilution range for all antimicrobial compounds tested was from 256 to 0.00003 μg/mL.

Biofilm production was tested by fluorescent *in situ* hybridization, as previously described [16], in two broth media, TSB (Tryptic Soy Broth, Oxoid, CM0129B) and BHIB (Brain Heart Infusion Broth, Oxoid, CM0225), using the universal bacterial probe, Eub338, labelled with fluorescein (Stabvida, Portugal). Wilcoxon signed ranks test was applied for statistical purposes.

From the 66 UPEC dog isolates evaluated, 31 isolates were biofilm-positive in BHIB at 24 hours, 51 at 48 hours, and 59 at 72 hours. In TSB, a higher number of biofilm-producing isolates was observed at all incubation times: 35 isolates at 24 hours; 52 at 48 hours; 62 at 72 hours.

No significant differences (P > 0.05) were found between biofilm formation in the two culture media, but significant differences were found between biofilm production between 24 and 48 hours, 48 and 72 hours, and 24 and 72 hours (P < 0.05).

Association between biofilm formation in TSB at 24 hours and the presence of *cnf1*, *hly*, *pap* and *sfa* was not significant (P > 0.05), whilst there was a significant association between biofilm and *afa* and *aer* (P < 0.05) (Table 1). Biofilm production was also associated to the presence of the β-lactamase genes *bla*_TEM*,*_*bla*_OXA-1,_*bla*_SHV_ and *ampC* (P < 0.05) (Table 1)*.*

**Table 1:** **Biofilm production and virulence and β-lactamase genes presence in 66 *E. coli *** **isolates from dogs with urinary infections**^**a**^

	**Biofilm production**	**Virulence genes**						**Multiplex-PCR**				
**Isolate**	**TSB, 24 hours**	***pap***	***sfa***	***afa***	***hly***	***cnf1***	***aer***	**TEM**	**SHV**	**OXA**	**AMP C**	**CTX-M**
5	Negative	-	+	-	-	-	-	-	-	-	-	-
13	Negative	-	+	-	-	-	-	-	-	-	-	-
21	Negative	+	+	+	+	+	-	-	-	-	-	-
34	Negative	-	-	-	-	-	-	-	-	-	-	-
36	Positive	-	-	-	-	-	-	-	-	-	-	-
43	Negative	-	-	-	-	-	-	-	-	-	-	-
78	Positive	+	-	-	-	-	+	+	-	-	+	-
84	Positive	-	+	-	+	+	-	-	-	-	-	-
88	Negative	+	+	-	+	+	-	-	-	-	-	-
91	Negative	-	-	-	-	-	+	+	-	-	+	-
95	Negative	-	-	-	-	-	+	+	-	-	+	-
99	Positive	-	-	-	-	-	-	-	-	-	-	-
109	Positive	+	+	-	+	+	-	-	-	-	-	-
115	Positive	-	+	-	-	-	-	-	-	-	-	-
125	Positive	-	-	-	-	-	-	+	-	-	+	-
128	Negative	-	-	-	-	-	-	-	-	-	-	-
133	Positive	-	+	-	-	-	-	-	-	-	-	-
134	Positive	-	+	-	-	-	-	-	-	-	-	-
138	Positive	+	-	-	-	-	+	-	-	-	-	-
174	Negative	-	+	-	-	-	-	-	-	-	-	-
179	Negative	-	+	-	-	-	-	-	-	-	-	-
188	Negative	-	-	-	-	-	+	+	-	-	+	-
194	Negative	-	-	-	-	-	+	+	-	-	+	-
207	Negative	+	+	-	+	+	-	-	-	-	-	-
209	Negative	+	+	-	+	+	+	-	-	-	-	-
224	Negative	-	-	-	-	-	+	+	-	-	+	-
226	Negative	-	-	-	-	-	+	+	-	-	+	-
227	Positive	-	+	-	+	+	-	-	-	-	-	-
237	Negative	+	+	-	+	+	+	-	+	-	+	-
238	Positive	+	+	-	+	+	+	-	+	-	+	-
239	Positive	+	+	-	+	+	+	-	+	-	+	-
250	Negative	+	-	-	-	-	-	+	-	-	+	-
251	Positive	+	+	-	+	+	-	-	-	-	-	-
257	Negative	+	+	-	+	+	-	+	-	-	+	-
258	Negative	-	-	-	-	-	-	+	-	-	+	-
271	Positive	-	-	-	-	-	-	+	-	-	+	-
274	Positive	-	-	-	-	-	+	+	-	-	+	-
291	Positive	+	+	-	+	+	-	+	-	-	+	-
304	Positive	-	+	-	-	-	-	-	-	-	-	-
320	Negative	-	-	-	-	-	+	+	-	-	+	-
325	Positive	+	+	-	-	+	+	-	-	-	-	-
327	Positive	+	-	-	+	+	-	-	-	-	-	-
347	Negative	-	+	-	+	+	+	-	-	+	+	-
354	Positive	-	-	-	-	-	+	-	-	+	+	-
372	Negative	+	+	-	+	+	-	-	-	-	-	-
374	Negative	+	+	-	+	+	-	-	-	-	-	-
386	Positive	-	-	+	+	-	-	+	-	-	+	-
401	Positive	+	+	-	+	+	+	-	-	-	-	-
403	Positive	-	+	-	-	-	-	+	-	-	+	-
417	Negative	-	-	-	-	-	-	-	-	-	-	-
443	Positive	+	+	-	+	+	-	-	-	-	-	-
449	Positive	-	+	-	-	-	-	-	-	-	-	-
457	Negative	+	+	-	+	+	-	-	-	-	-	-
461	Positive	+	+	-	+	+	-	-	-	-	-	-
483	Positive	-	+	-	-	-	-	-	-	-	-	-
488	Negative	+	+	-	+	+	-	-	-	-	-	-
505	Negative	-	-	-	-	-	+	+	-	-	+	-
528	Positive	-	-	-	-	-	-	-	-	-	-	-
531	Positive	+	+	-	+	+	+	-	-	-	-	-
536	Positive	-	+	-	-	-	-	-	-	-	-	-
539	Positive	-	-	-	-	-	+	+	-	-	+	-
540	Positive	+	+	-	+	+	-	-	-	-	-	-
553	Negative	-	-	-	-	-	-	+	-	-	+	-
554	Positive	+	+	-	+	+	+	-	-	-	-	-
560	Positive	+	+	-	+	+	-	-	-	-	-	-
566	Negative	+	-	-	-	-	+	-	-	-	-	-
Total (n=)	35	28	38	1	28	27	23	20	3	2	25	0
%	53.0	42.4	57.6	1.5	42.4	40.9	34.8	30.3	4.5	3.0	37.9	0

^a^Virulence and β-lactamase genes results are adapted from Féria *et al*. [12] and Pomba *et al*. [13]; **+** PCR positive result; **−** PCR negative result.

Fluoroquinolones resistance is summarized in Table 2. Resistance was found in 13.6% of the uropathogenic isolates (n = 9) towards ciprofloxacin, enrofloxacin, marbofloxacin and orbifloxacin. One additional isolate was resistant to orbifloxacin. All *E. coli* isolates were simultaneously resistant to all the fluoroquinolones tested.

**Table 2:** **Antimicrobial susceptibility to 2**^**nd **^**generation fluoroquinolones and its relation with biofilm production in 66 *E. coli *** **isolates from dogs with urinary infections**

		**Fluoroquinolones**			
		**CIP**	**ENR**	**MAR**	**OBX**
Susceptibility criteria (μg/mL)^a^	Susceptible; Resistant	≤ 1; ≥ 4	≤ 0.5; ≥ 4	≤ 1; ≥ 4	≤ 1; ≥ 8
Susceptibility results	Susceptible	57 (86.4%)	57 (86.4%)	57 (86.4%)	56 (84.8%)
	Resistant	9 (13.6%)	9 (13.6%)	9 (13.6%)	10 (15.2%)
Relation with biofilm production		P < 0.05	P < 0.05	P < 0.05	P < 0.05

^a^Isolates that were categorized as intermediate to the antimicrobial compounds were recorded as susceptible, since the bactericidal activity of these antimicrobials depends of their *in vivo* concentration and quinolones are known to be concentrated from 100- to 300-fold within the urinary tract (Cohn and others 2003); CIP Ciprofloxacin (CLSI 2014), ENR Enrofloxacin (CLSI 2013), MAR Marbofloxacin (CLSI 2013), OBX Orbifloxacin (CLSI 2013).

Biofilm formation has been described as an important *E. coli* virulence factor in human UTI. In this study, biofilm-forming ability of 66 UPEC dog isolates was evaluated. Previous works showed that isolates ability to form biofilm depends upon the medium used and time of observation [16-19]. In our study, no differences were found regarding biofilm production in BHIB and TSB. Almost half of the isolates were able to form biofilm at 24 hours in both media, and this percentage significantly increased with incubation time.

Association between biofilm and other virulence factors has already been studied [18]. In this work, biofilm was not associated to toxin production (*hly* and *cnfI*), or to filamentous adhesions involved in host specific adhesion (*sfa* and *pap*). Nevertheless, associations between biofilm and *afa* and *aer* were significant. These results may indicate that adhesive non-fimbrial adhesions are important for the initial steps of biofilm formation and that the aerotaxis receptor may be involved in the oxygenation of these structures. Biofilm production was also associated to the presence of the β-lactamase genes*.* Our results are not in accordance with previous works [18,20] that stated that *E. coli* strains that are β-lactamase producers may not be able to form biofilms*.*

Regarding fluoroquinolones resistance, compounds tested showed an *in vitro* efficacy of more than 80%, as already observed by other authors [11,21]. It is important to refer that although these broad-spectrum antibiotics are extensively used for treatment of animal related infections, their efficacy remains high [11].

Biofilm structures are believed to impair antimicrobial compounds action [10,22]. Association between biofilm and fluoroquinolone resistance was considered significant in all time points, independently of the media, which is in agreement with previous human UTI studies [9]. Biofilm formation by UPEC may contribute for UTI treatment failure in dogs, since these structures are responsible for the establishment of bacterial reservoirs inside the bladder cells, allowing them to overcome the host immune defences and to establish recurrent infections [9].

To our knowledge, this is the first report of the association between biofilm formation and fluoroquinolone resistance in *E. coli* dog UTI isolates, representing an important novelty. This fact is relevant for biofilm and antimicrobial resistance control in veterinary medicine and the establishment of more adequate therapeutic protocols.

### Animal ethics

No experimental research on vertebrates or any regulated invertebrates were performed in this study.

## Competing interests

The authors declare that they have no competing interests.

## Authors’ contributions

MO participated in the study conception and design, carried out the biofilm studies and drafted the manuscript. CP participated in the study conception and design, carried virulence and antimicrobial resistance genes studies and minimum inhibitory concentration determinations and helped to draft the manuscript. FRD participated in the biofilm studies. All authors read and approved the final manuscript.
